# Influence of silane and heated silane on the bond strength of lithium disilicate ceramics - An in vitro study

**DOI:** 10.12669/pjms.323.9851

**Published:** 2016

**Authors:** Tariq Abduljabbar, Mohammed Ayedh AlQahtani, Zaid Al Jeaidi, Fahim Vohra

**Affiliations:** 1 Dr. Tariq Abduljabbar, Associate Professor, Department of Prosthetic Dental Sciences, College of Dentistry, King Saud University, Riyadh, Saudi Arabia; 2 Dr. Mohammed Ayedh AlQahtani, Lecturer, Department of Prosthetic Dental Sciences, College of Dentistry, King Saud University, Riyadh, Saudi Arabia; 3 Dr. Zaid Al Jeaidi, Conservative Dental Science Department, College of Dentistry, Prince Sattam Bin Abdulaziz University, Al-Kharj, Saudi Arabia; 4 Dr. Fahim Vohra, Associate Professor, Department of Prosthetic Dental Sciences, College of Dentistry, King Saud University, Riyadh, Saudi Arabia

**Keywords:** Silane, Lithium disilicate, Silane heat treatment, Microtensile bond strength

## Abstract

**Objectives:**

To evaluate the effect of silane application and silane heat treatment on lithium-disilicate ceramic when bonded to composite resin.

**Methods:**

Twelve blocks of lithium-disilicate (LD) ceramic were fabricated and bonding surfaces were etched using 9.5% hydrofluoric acid (90 seconds). Three experimental groups resulted from the various surface treatment combinations, which included, no silane application (NS) (controls), silane application (S) and silane heat treatment (HS) (100°C for 5 minutesutes). Ceramic and composite resin blocks were bonded using an adhesive resin and light cured restorative composite as a luting agent, under standard conditions. A total of 90 specimen sticks (8 x 1mm²) were subjected to micro-tensile bond strength testing. The means of micro-tensile bond strength (µ-tbs) of the study groups were analyzed using t-test and ANOVA. The tested specimens were analyzed for mode of failure using scanning electron microscopy (SEM).

**Results:**

The highest µ-tbs value (42.6 ±3.70 MPa) was achieved for LD ceramics with heat-dried silane. Both silane application and heat treatment of silane resulted in significant (p<0.05) improvements in micro-tensile bond strength of LD ceramics when bonded to resin composite.

**Conclusions:**

The application of silane and its heat treatment showed significant improvement in bond strength of lithium disilicate ceramic when bonded to composite.

## INTRODUCTION

Development of resin-bonded all-ceramic restoration has led to their extensive use as veneers, crowns, inlay and onlays. A glass ceramic based on lithium disilicate (LD) (SiO2–Li2O) crystals has been developed to extend the use of resin-bonded ceramic restorations for bridge construction. This pressed glass-ceramic has an improved flexural strength and fracture toughness as compared to others (leucite reinforced ceramics) and demonstrates abrasion resistance, chemical durability and optical properties well within the dental standards.^1^ Furthermore it has gained support for its use in fabrication of 3-unit bridges for posterior region up to the second premolar.^1^-^3^

The standard regime for conditioning teeth is etch-prime-bond. In a similar way, the internal surface of the ceramic restoration must be prepared to optimize the bond between the ceramic and resin. For the optimization of micromechanical bond hydrofluoric acid (HF acid) has been the preferred acid treatment for glass ceramics.^4^-^6^ In addition to this mechanically retentive surface, the application of a silane provides an effective and durable chemical bond.^7^,^8^ In addition, thermal treatment of silanes have been employed for improvements in ceramic bonding.^9^ Although many authors consider treatment of ceramic surface both with HF acid and silane as indispensable.^4^,^10^ Effect of silane application and silane heat treatment in adhesive bonding of lithium disilicate ceramics has not been assessed. It is hypothesized that heat treatment after silane application would improve the bond strength of lithium disilicate ceramics. Therefore, the present study was aimed to evaluate the influence of silane heat treatment on the microtensile bond strength (μ-tbs) of lithium disilicate ceramics.

## METHODS

Twelve ceramic blocks of (4x6x8 mm) using lithium disilicate ceramic (LD) (E-max Press®, Ivoclar Vivadent, AG, Schaan / Liechtenstein) was fabricated using lost wax technique and compatible hot pressing furnaces. After removal from investment, the interaction layer was removed from the cast ceramic blocks by grit blasting with 70µm glass beads, these were further polished from 240 to 1200 grit SiC abrasive coated paper and finished with 1 µm aluminutesa. All blocks were ultrasonically (Fine Sonic US Cleaner, DiaDent, BC, Canada) cleaned with distilled water for 10 minutes after polishing.

All the ceramic blocks were etched using 9.5% HF acid (Ceram Etch, Gresco product Int., Stafford, TX) for 90 seconds and rinsed with water for 20 seconds (sec) for removal of HF acid. The etched and rinsed blocks of ceramic were exposed to ultrasonic cleaning for five minutes in distilled water bath. The following surface treatments were applied to the ceramic blocks:

**Group A:** No application of silane (Calibra, Silane Coupling Agent, Dentsply, Caulk, Surrey, United Kingdom). (NS).

**Group B:** Silane application with air drying (S).

**Group C:** Silane applied and heat dried (5 minutes at 100ºC in hot air oven) (HS).

An adhesive (Optibond FL, Kerr Dental, Orange, CA, USA) was applied to all the surface treated ceramic blocks. Composite blocks (Hybrid filler, Filtek Z350 XT, 3M ESPE, St. Paul, MN, USA) (4x6x8 mm) were fabricated using a rubber (Aquasil, Putty, Dentsply, Surrey, United Kingdom) copy mould of ceramic blocks. The composite and ceramic blocks were bonded using a customized verticulator, at a 10 seconds load application of one kilogram, light cured (LED) for 160 seconds with an intensity of 650 mWcm-². A slow speed diamond wheel saw (Isomet 1000, Buehler, Lake Bluff, IL, USA) was used to section the composite-ceramic blocks at a constant speed of 500 rpm at 250 grams force. All specimen sticks of 1mm² cross section each were stored for 24 hours in normal saline at 37°C. Thirty specimen for each group were randomly selected for microtensile testing. The specimens were attached to the tester jaws using cyanoacrylate adhesive (Zapit, Dental ventures Inc, CA, USA) and loaded to failure under tension at a crosshead speed of 0.5mm/minutes using a microtensile tester (Bisco Inc., Virginia, USA). The means of μ-tbs were analyzed using t-test and ANOVA. The materials and equipment used in the methodology of the study are detailed in Appendix A.

Randomly selected four fractured pairs of each group were further used for fractographic analysis using a scanning electron microscope (SEM) (XL 30CP, Phillips, MA, USA). The mounted and alcohol wiped specimens were sputter coated with gold for 180 seconds at 40mA, creating a 30nm thick layer. This was examinutesed under different standard magnifications of SEM operated at 20KV using secondary electron detection, by single operator. Fractographic analysis was based on the following categories. (1) Failure at the bonding material and ceramic interface (2) Failure at resin/ceramic interface, progressing into adhesive resin. (mixed failure) (3) Adhesive failure fracture at the bonding material / composite interface. (4) Cohesive failure within the cement or the ceramic or composite materials.

## RESULTS

All data passed the normality test using the Kolmogorov and Smirnov (KS) test. The surface area of the bar specimens as recorded at the interface region was 1.0 mm^2^ (SD 0.105), without any statistical significant difference (P=0.11). Silane application and heat treatment of silane showed statistically significant effects on the μ-tbs. The lowest μ-tbs value was obtained for Group A, with no silane application (NS) 34.95(±3.12) Mpa. The highest μ-tbs value was obtained for group C with heat treatment of silanes at 42.6 (±3.70) MPa the means and standard deviations of µtbs achieved in each of the experimental groups are summarized in Table-I. Analysis of variance revealed statistically significant differences in bond strengths between the experimental groups (P < 0.01). (Table-I) The maximum average difference in means was due to silane application (group A vs group B, 4.983 MPa, P<0.001) followed by the effect of silane heat treatment (group B vs group C, 2.69 MPa, P<0.05).

**Table-I:** Comparison of means and standard deviations of micro tensile bond strength of experimental groups using ANOVA.

*Ceramic type*		*Study groups*		

		*Group A*	*Group B*	*Group C*	*P value*
Lithium disilicate ceramic (µtbs)	Mean	34.95	39.94	42.63	< 0.01^*†^
	SD	3.123	2.589	3.701	

* significant,

† Analysis of variance (ANOVA) was performed

SD: Standard deviation, µtbs: Microtensile bond strength.

**Fig.1:**
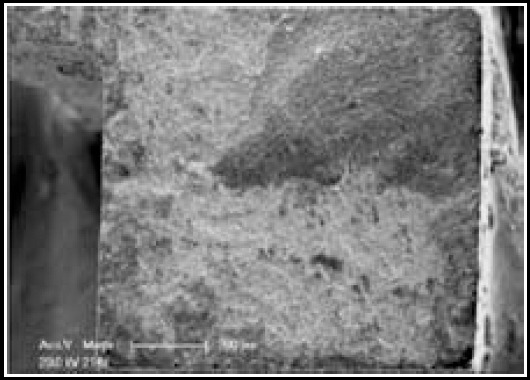
Example of category 2 failure propagating into resin at x214 magnification (Specimen from group C).

**Fig.2:**
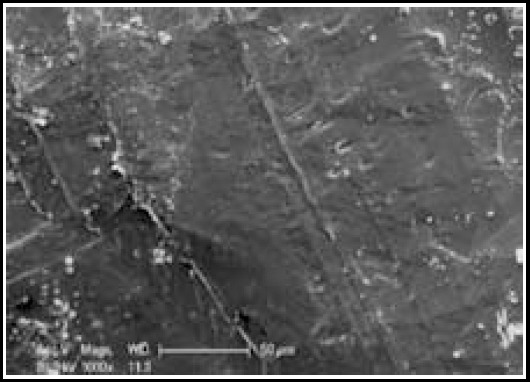
Example of category 1 failure at x1000 magnification (Specimen from group A).

The fractured surfaces were observed under scanning electron microscope to determinutese the mode of failure based on the origin and location of fracture. 50% and 75% of specimens in group B and group C showed category two failures i.e. failure at resin/ceramic interface, progressing into adhesive resin, respectively. (Fig.1) All specimens in group A showed adhesive failures at the resin/ ceramic interface. (Fig.2)

**Table-II:** Percentage distribution of failure modes in the experimental groups.

*Mode of failure*	*Group A*	*Group B*	*Group C*
Category 1	100	50	25
Category 2	0	50	75
Category 3	0	0	0
Category 4	0	0	0

## DISCUSSION

Despite the successful introduction of ceramic core systems, bondable ceramic restorations in the form of inlays, onlays, veneers and crowns still form a major part of conservative aesthetic prosthodontics. The ability of these restorations to create a predictable bond with the tooth is the most critical factor in their success.^11^,^12^ Therefore the experiment in this study involves evaluating the influence of silane application and heat treatment of silane on microtensile bond strength of ceramics. Resin composite was used as the bonding substrate, firstly because the aim was to evaluate contribution of surface treatment steps of ceramics. Secondly, in order to minutesimize the likely variables in the experiment e.g. human dentine, quantity and structure of exposed dentine, size and number of tubuli openings and surface treatment.^13^ In the study, lithium disilicate based ceramic was also evaluated for surface treatment effects. As it has been advocated to be used for three unit bridges^2^,^3^ due to its unique ability of adhesive bonding and having mechanical properties (flexural strength and fracture toughness) superior to other bondable ceramics (Etchable ceramics).

Hydroflouric acid (HF) etch was used as a constant i.e. all groups received HF acid treatment. HF acid etching of ceramics produces a consistent and favourable micro morphology of surface for micromechanical retention for both the ceramics used in the study.^14^ It further reduces the surface contact angle, increasing the surface free energy and wettability of the luting agent.^15^,^16^ Ninety seconds was considered the optimum time for HF acid etching as it is reported that continued and frequent HF acid application results in reduction of flexural strength of lithium disilicate ceramics.^17^ A microtensile bond strength test was used as it represents the true adhesive bond strength^18^ compared to shear bond test which reflects the strength of base material.^19^ A non-trimmimg technique for specimen production was employed as less stresses are introduced at the interface.^20^

The most important surface treatment factor affecting the bond strengths of LD ceramics was the application of silane. One of the previous report^7^ have identified similar outcomes rendering silane as having a major effect on bonding of resin to lithium disilicate ceramics. Silanes being bifunctional promote ceramic resin adhesion and facilitate resin penetration into the acid etched ceramic by enhancing the wetting of the surface. In addition, the heat treatment of silane also revealed significant improvements in bond strengths. Silane when applied on the ceramic surface gives a layered configuration.^21^,^22^ Heat treatment at 100°C has been shown to merge the layered surface, removing the interphase and increasing the bond strength of composite to ceramic.^23^ An increase in bond strengths using heated two bottle silanes has been reported,^9^ however not in case of prehydrolized silanes used in present study. Therefore this is the first study reporting the improvements in bond strengths of LD ceramics after heat treatment of prehydrolized silanes.

The quality of the bond should not be assessed based on bond strength data alone. 50% and 75% of failures in specimen with silane application (group B) and silane heat treatment (group C), respectively showed category two failures i.e. failure at resin/ceramic interface, progressing into adhesive resin, respectively propagating into the luting resin, this reflects the increase in bond strength values shown through micro-tensile bond strength testing.

In light of the findings in the present study, it is clinically recommended that lithium disilicate ceramic restorations prior to adhesive bonding should receive silane application along with its heat treatment for optimum adhesive ceramic bonding. In addition further studies assessing the clinical contaminutesation of ceramic surface and effect of silane and HF acid on their bond strength are recommended.

## CONCLUSION

Within the limitations of this in-vitro study, it is concluded that the application of prehydrolized silane and its heat treatment showed significant improvement in bond strength of lithium disilicate ceramic when bonded to composite.

## Appendix

**Table:**

Appendix A: Materials and Equipment Used
E-max Press® (E): (lithium disilicate ceramic, Ivoclar Vivadent, MO2 lot no. 4312
9.5% Hydroflouric acid: Ceram Etch, Gresco product Int
Silane (S): Calibra, Silane Coupling Agent, Dentsply, Caulk. Lot no.070307)
Composite Blocks And luting agent: Filtek Z350 XT, Hybrid filler particles, Universal Restorative, 3M ESPE, Shade A1E, Lot no. 8CU
Aquasil soft putty: Polyvinylsiloxane impression material, Caulk, Dentsply
Cyanoacrylate adhesive: Zapit, Dental ventures Inc, CA, USA
Curing light: Bluephase ® C8, Ivoclar Vivadent, AG, Schaan / Liechtenstein
Hot pressing furnaces: Multimat 2 (Dentsply) and Programat P300 (Ivoclar Vivadent, AG, Schaan / Liechtenstein)
SiC abrasive coated paper & Aluminutesa: Mark V Laboratory, East Granby, CT, USA
Isomet 1000: Buehler, Lake Bluff, IL, USA
Microtensile tester: Bisco Inc., Virginia, USA.
Scanning electron microscope: XL 30CP, Phillips
